# Eating behaviour and eating disorders in individuals with rare neurodevelopmental variants: current knowledge and future research directions

**DOI:** 10.3389/fpsyt.2025.1627378

**Published:** 2025-10-20

**Authors:** Samuel J. R. A. Chawner

**Affiliations:** Centre for Neuropsychiatric Genetics and Genomics, Cardiff University, Cardiff, United Kingdom

**Keywords:** eating disorders, eating behaviour, psychiatric genetics, copy number variant (CNV), medical genetics

## Abstract

Rare neurodevelopmental copy number variants (ND-CNVs) have been implicated in a range of psychiatric and neurodevelopmental conditions. Despite their known association with a range of behavioural outcomes, the role of ND-CNVs in eating disorders and related traits remains underexplored. This perspective synthesises current knowledge on the association between ND-CNVs, eating disorders and eating behaviour, highlighting the potential for research into ND-CNVs to provide insights into the genetic architecture of eating disorders. Initial CNV genome-wide association studies have been conducted for anorexia nervosa, and there is now a need to investigate the roles of ND-CNVs in larger samples and across a range of eating disorders. Population cohort studies, and genetic-first designs whereby individuals with a clinical genetic diagnosis undergo deep phenotyping, provide strong evidence for the impact of ND-CNVs on body mass index (BMI), with some ND-CNVs associated with increased BMI, and others decreased BMI relative to the population. Although there have been detailed characterisations of eating behaviour phenotypes in Prader-Willi Syndrome and 16p11.2 Deletion and Duplication Syndromes, overall population and genetic-first studies of the impact of ND-CNVs on eating behaviour and eating disorder risk have been limited. Key research gaps to overcome include the lack of relevant eating disorder phenotype data in large-scale cohorts, limited research into the mechanistic pathways between genotype and phenotypic outcome, and the need for research to include diverse populations. Cross-disciplinary collaboration will be essential to advance the field to enable the development of effective interventions and genetic counselling for eating behaviour and eating disorders.

## 1 Introduction

A number of rare genomic conditions, including recurrent pathogenic copy number variants [CNVs, deletions and duplications >1000 base pairs (1)], have been identified to confer risk for neurodevelopmental and psychiatric conditions (ND-CNVs) including intellectual disability (ID), attention deficit hyperactivity disorder (ADHD), autism and schizophrenia (2–6) (see **Table 1**). CNVs are increasingly being detected in clinical settings through the use of technologies including chromosomal microarray allowing identification of sub-microscopic CNVs [resolution of ~50–100 kilobase pairs (7)] which would have been undetected under methods such as karyotyping (8–10). Furthermore, the introduction of exome and whole genome sequencing in clinical genetic testing has significantly increased diagnostic yield (11) and improved resolution to that of a single base pair, allowing for the diagnosis of pathogenic sequence variations within a single gene, including intragenic CNVs, single-exon changes and point mutations (8, 12, 13). The most frequently studied CNVs are recurrent CNVs which are predominantly mediated by non-allelic homologous recombination, which occurs between highly homologous low copy repeats, resulting in segmental deletions or duplications (3, 14–16). Non-recurrent variants typically occur at low frequencies, posing challenges for research focussing on the effects of specific variants (17). Consequently, the majority of CNVs examined in research, and those discussed in this perspective, are recurrent CNVs. In contrast, non-recurrent CNVs arise at variable genomic loci with heterogeneous breakpoints. Recurrent CNVs, by comparison, tend to recur at specific genomic regions across individuals, leading to higher population frequencies. For example, analysis of 12,252 parent-offspring trios from the Norwegian Mother, Father, and Child Cohort Study (MoBa) estimated the prevalence of 26 recurrent CNVs in live-born children at 0.48%, with individual variants ranging from 0.008% to 0.05% (18).

**Table 1:** Frequent CNVs associated with risk for Neurodevelopmental disorders (NDDs).

Locus	Rearrangements	Syndrome	Position of critical region Hg19	Key genes
1q21.1 proximal	del and dup	Thrombocytopenia-Absent Radius syndrome (del)	chr1:145,394,955-145,807,817	*RBM8A, PDZK1P1*, *GPR89C*
1q21.1 distal	del and dup		chr1:146,527,987-147,394,444	*HYDIN2*, *PRKAB2*, *CHD1L*, *BCL9*, *GJA5*, *GJA8*, *GPR89B*
2p16.3	del		chr2:50145643-51259674	*NRXN1*
3q29	del		chr3:195,720,167-197,354,826	*DLG1*
7q11.23	del and dup	Williams-Beuren Syndrome (WBS) (del)	chr7:72,744,915-74,142,892	*CLDN3, CLDN4, GTF2, ELN, LIMK1, KCTD7, CLIP2, STX1A*,
9q34	del	Kleefstra Syndrome (del)	chr9:140,513,444-140,730,578	*EHMT1*
15q11.2	BP1-BP2; del and dup		chr15:22,805,313-23,094,530	*CYFIP1*
15q11-q13	BP3-BP5; del and dup	Prader-Willi Syndrome (del) & Angelman Syndrome (dup)	chr15:29,161,368-32462776	*UBE3A, ATP10A, GABARB3, GABARA5, GABARG3*
15q13.3	del and dup		chr15:32,017,070-32,453,068	*CHRNA7*
16p13.11	del and dup		chr16:15,511,655-16,293,689	*NDE1, MYH11*
16p11.2 proximal	del and dup		chr16:29,650,840-30,200,773	*KCTD13, ALDOA, CORO1A, MAPK3, TAOK2*
16p11.2 distal	del and dup		chr16:28,823,196-29,046,783	*TUFM*
17q12	del and dup	Renal cysts and diabetes syndrome (RCAD) (del)	chr17:34,815,904-36,217,432	*NF1*
22q11.2	del and dup	DiGeorge Syndrome, Velocardiofacial Syndrome and 22q11.2 Deletion Syndrome (del)	chr22:19,037,332-21,466,726	*TBX1, COMT, PI4KA,SEPT6*
22q13	del and dup	Phelan-McDermid Syndrome (PMDS) (del)	chr22:51113070-51171640	*SHANK3*

Although individually rare, collectively, neurodevelopmental variants have been implicated in ~15-40% of patients with neurodevelopmental conditions (5, 8), and in 5% of individuals with schizophrenia (19), rising to 8% for child-onset schizophrenia (20). Although these rare variants are strongly associated with psychiatric conditions, they have incomplete penetrance and exhibit a high degree of pleiotropy, conferring risk for a broad range of psychiatric symptomatology, cognitive deficits, and medical/physical comorbidities across the lifespan (14, 21–25). The study of ND-CNVs has provided valuable insights into the aetiology of psychiatric conditions and highlighted the overlap between neurodevelopmental conditions and schizophrenia. The identification of ND-CNVs has paved the way for genetic-first studies, where children with risk variants are prospectively assessed throughout development (26, 27). Additionally, genetic-first animal and cellular models offer insights into the mechanisms by which genomic risk for psychiatric outcomes manifests at cellular and neurobiological levels (28, 29). However, there has been a relative lack of research into the impact of rare ND-CNVs on eating disorders and related traits, limiting our understanding of the genetic architecture and biological mechanisms underpinning these conditions.

It is important that the lack of research into the role of ND-CNVs in eating disorders and eating behaviour is addressed. In this perspective the term eating disorders is used this refers to those defined within the DSM-5 criteria including anorexia nervosa (AN), bulimia nervosa (BN), binge-eating disorder (BED), avoidant restrictive food intake disorder (ARFID), and pica (30). Across medicine, the study of rare variants has provided transformative insights into the biological mechanisms underlying health conditions. Within the psychiatry field, schizophrenia risk CNV regions have been found to be enriched within genes involved in inhibitory GABAergic (gamma-aminobutyric acid-ergic) and excitatory glutamatergic systems providing causal insights into schizophrenia pathogenesis (31). Furthermore, the identification of obesity-risk rare variants, including mutations in the leptin receptor gene (LEPR) (32) as well as POMC (33) and MC4R (34) genes, has highlighted that the leptin–melanocortin pathway is a key appetitive control circuit (35). It is not known the extent that rare genetic variation in genes influencing appetitive control contribute to eating disorders. Though it should be noted that eating disorders and Body Mass Index (BMI) overlap in common genetic risk, BMI polygenic risk score positively correlates with BN and BED, whereas for AN the direction of association was reversed (36). It is not known the extent that loci of rare variants identified for obesity also contribute to eating disorders.

Further evidence supporting the importance of research into the role of ND-CNVs in eating disorders and behaviours comes from the studies highlighting the significant genetic basis of these traits. Twin studies have highlighted that eating disorders have a significant genetic component, including 0.79 heritability for ARFID, 0.48-0.74 for AN, 0.55-0.62 for BN, and 0.39-0.45 for BED (37, 38). Furthermore high heritability has been found for eating disorder related traits including food fussiness 0.74-0.79 (39–41), appetitive traits 0.53-0.84 (42), and 0.61-0.80 for BMI (43). The significant genetic basis of eating disorders and related traits warrants future research identifying the specific risk genetic variants underlying heritability. Identification of rare genetic variants for eating disorders would also be a first step for elucidating the genomic relationships between eating disorders and other psychiatric conditions. For example, evidence that schizophrenia risk CNVs are also associated with neurodevelopmental conditions has highlighted the shared aetiology of these conditions supporting a neurodevelopmental hypothesis of schizophrenia aetiology (2, 44, 45). There is growing awareness of the clinical overlap between eating disorders and autism (46–48), and initial studies indicate shared common genetic risk factors between neurodevelopmental conditions and eating disorders including AN (49) and ARFID (50), but there is a lack of research into the contribution of rare variants to the shared aetiology of eating disorders and neurodevelopmental conditions. Together these different lines of evidence indicate that ND-CNVs are likely candidates as risk factors for eating disorders, and the next section outlines what is currently known about the association of ND-CNVs with eating disorders and eating behaviour.

## 2 Current knowledge of the association between ND-CNVs, eating disorders and related traits

### 2.1 Genome-wide association studies

Genome-wide copy number variation association studies (CNV-GWAS) of eating disorders have provided mixed findings. An early study reported one out of 109 individuals with AN carried an atypical 136 kb duplication that encompassed the SPN and QPRT genes (51). An early case-control study implicated the 13q12 region in AN (1033 AN cases), but there was not an overrepresentation of large rare CNVs in individuals with AN compared to controls (52). A larger study of 1,983 females with AN from the Genetic Consortium for AN (GCAN), found previously established ND-CNVs were present in AN cases (53), and one case had a 13q12 deletion replicating the previous study. The largest and most recent case-control CNV-GWAS of AN, with a sample size of 7414 AN cases and 5044 controls, found 21 nominally associated CNV regions that contribute to AN risk but none of the well-established syndromic ND-CNVs had a significant association with AN status (54). However CNVs in individuals with AN were found to be enriched in genes involved in synaptic function, metabolic and mitochondrial factors, and lipid characteristics, consistent with the metabo-psychiatric conceptualisation of the disorder (54, 55). There was also no evidence in this study of a global enrichment of rare CNVs in AN, and the contribution of ND-CNVs was limited in comparison to conditions such as schizophrenia (54). This could perhaps indicate the magnitude of contribution of rare variants to AN is lower than conditions such as schizophrenia, and therefore larger sample sizes may be needed for rare variant discovery for AN compared to schizophrenia. Though these findings should not necessarily be generalised to all eating disorders, as there has been a lack of CNV studies for other eating disorders including ARFID and pica, which have been under researched for genetic aetiology. Indeed for ARFID, it has been hypothesised that ND-CNVs may play a role due to the condition’s overlap with neurodevelopmental conditions (50, 56), similarly pica overlaps with neurodevelopmental conditions (57, 58) and therefore ND-CNVs may also contribute to the aetiology of pica. Although the AN CNV-GWAS studies described represent the largest to date, sample size lags behind that for other psychiatric conditions (schizophrenia n = 76,755, major depressive disorder n = 688,808) reducing power for gene discovery, particularly for identification of risk rare variants which requires large sample sizes simply to observe a variant with low population frequency (59). The potential for gene discovery for a given diagnosis is also influenced by the extent of genetic contribution to its aetiology. Therefore, for conditions like ARFID, which exhibit high heritability, there is substantial promise for identifying novel genetic risk factors (37). In contrast to the eating disorder field, there have considerable advances in the understanding of the genetic architecture of obesity, including CNVs (including 1p31.1 deletion, 16p12.3 deletion and 16p11.2 deletion) (35, 60), made possible due to the widespread inclusion of BMI phenotypes in large cohorts.

### 2.2 Population cohorts

Large-scale population cohorts have provided clear evidence of the role of ND-CNVs in extreme BMI outcomes, including both overweight and underweight outcomes, but there has been a lack of studies that have examined eating disorders and related eating behaviour traits. The impact of ND-CNVs on BMI has been demonstrated in the UK Biobank (61) cohort of 396,725 adults aged 40–69 recruited from the UK population, which found 13 ND-CNVs to be associated with increased BMI compared to adults without a ND-CNV, and 3 ND-CNVs were associated with decreased BMI. A meta-analysis of 191,161 adults from 26 cohorts revealed associations at 1q21.1, 7q11.23, 16p11.2, 18q21.32 22q11.21, with either BMI, weight, and/or waist–hip ratio (62). The impact of ND-CNVs on BMI leads to wide-ranging effects, including diabetes, and hypertension (63). In a case-only study the 16p11.2 deletion has a prevalence of 0.5% within a cohort of adult patients who underwent bariatric surgery (64). The lack of research into the impact of ND-CNVs on eating disorder risk in population cohorts is partly due to a lack of phenotype data in such cohorts, but one approach to overcoming this is to derive phenotypic proxies by developing algorithms that combine information from medical registry diagnoses and/or eating disorder-related questionnaire items (37, 65).

### 2.3 Clinical studies of ND-CNV carriers identified via clinical settings

Genetic-first studies, whereby individuals with ND-CNVs diagnosed within medical genetic clinics have undergone deep phenotyping protocols, have revealed variants associated with obesity including 16p11.2 Deletion and 22q11.2 Deletion (60, 66). 16p11.2 Duplication has been associated with failure to thrive in childhood and being clinically underweight in adulthood (51); and a high prevalence of nutritional problems and failure to thrive has been reported for 22q11.2 Duplication Syndrome (67). Genetic-first studies of psychiatric risk CNVs have examined a range of domains across childhood development (6, 24), but the majority have not included eating disorders and eating behaviour traits in their deep phenotyping protocols.

The ND-CNVs conditions which have been well-characterised for eating behaviour include Prader-Willi Syndrome (PWS, 15q11.2-q13 deletion) and reciprocal 16p11.2 Deletion and Duplications. Prader-Willi Syndrome (PWS) is a complex neurodevelopmental genetic condition resulting from absence of expression of imprinted genes in the paternally derived region of the chromosome 15q11.2-q13.1 (68). One of the hallmark features of PWS is hyperphagia, an intense and insatiable hunger that leads to chronic overeating and severe obesity (69). Individuals with PWS experience a persistent sensation of hunger and an extreme drive to consume food, often accompanied by food-related behavioural problems such as food-seeking and hoarding (70). Managing hyperphagia in PWS is challenging, strategies include strict supervision of food intake, creating a food-secure environment where access to food is controlled, and behavioural and pharmacological interventions to address food-related behaviours (69). In the last couple of decades, the introduction of microarray testing in clinical settings has led to the identification of 16p11.2 Deletion and 16p11.2 Duplication variants. The 16p11.2 locus is of great interest as reciprocal genetic changes lead to a “mirror” phenotype (51) whereby carriers of the 16p11.2 deletion display a penetrant form of obesity (OR = 43) (60) and are known to exhibit hyperphagia (71) and emotional over-eating (72), whereas duplication carriers are at increased risk of being chronically underweight and have been reported to show restrictive eating behaviour and heightened responsiveness to satiety (51, 72). This mirror phenotype highlights the importance of gene expression at 16p11.2 on Body Mass Index (BMI) and eating behaviour, and potentially on eating disorder outcomes. The association of 16p11.2 with BMI, is a robust and replicable finding supported by genome wide association studies of BMI, and large population cohort and clinical studies (51, 60–62, 72). Studies of eating behaviour in 16p11.2 Deletion and Duplication indicates eating behaviour changes in terms of satiety responsiveness, food responsiveness, and emotional overeating (72), and cross-sectional evidence indicates that behavioural changes occur before later extreme BMI outcomes (72). Individuals with 16p11.2 deletion are more likely to engage in eating in the absence of hunger (EAH), where they consume food in response to external cues or boredom rather than physiological hunger (71). This disinhibited eating behaviour contributes to the development of obesity in individuals with 16p11.2 deletion. It has been investigated whether the EAH in individuals with 16p11.2 Deletion represents binge-eating, but an initial findings reported no loss of control eating in childhood indicating this eating behaviour does not meet BED criteria (71). However, these findings warrant replication and investigation across a range of ages.

It is important to recognise that the relatively detailed characterisation of eating behaviour in PWS and 16p11.2 variants, does not mean that other ND-CNVs do not necessarily impact eating behaviour. Rather the focus of the literature on these conditions is likely to represent historical reasons, PWS was first characterised in 1956 (73), and the 16p11.2 locus has received great attention following seminal work published in Nature describing the mirror effect reciprocal variants have on BMI (51). Indeed, studies of ND-CNVs in UK Biobank highlight that a range of other variants lead to a range of extreme BMI outcomes (61), and therefore may lead to similar eating behaviour outcomes.

## 3 Current research gaps

### 3.1 Phenotype bias

The majority of research of the role of ND-CNVs in eating disorders and related traits has predominantly centred on BMI and hyperphagia, with a relative paucity of studies on eating disorder and broader restrictive eating and avoidant eating behaviour phenotypes. Notably, there has only been a large-scale CNV-GWAS study for AN (54), and a lack of studies for other eating disorders including BN, BED, ARFID and pica (56). This bias stems from a general lack of research into eating disorders, driven by disparities in funding compared to other psychiatric conditions (74, 75). Within eating disorder research, initial work has primarily focussed on AN, with considerably less known about other eating disorders. The collection of large patient cohorts with eating disorders lags behind that for other psychiatric conditions. However, there are now concerted efforts to accelerate research on ARFID, BN and BED (76, 77). Historically, population cohorts have lacked detailed phenotype data, but the retrospective derivation of variables using algorithm approaches combining medical records and questionnaire data and the addition of relevant measures in cohorts will expand research possibilities. Leveraging existing population cohort and consortium infrastructures to enrich for eating behaviour and eating disorder measures will be crucial. Large-scale consortia approaches have been beneficial for the study of schizophrenia development in 22q11.2 Deletion Syndrome (78), and demonstrate what may be possible for studies of individuals at high genomic risk for eating disorders.

### 3.2 Lack of mechanistic insights – deep phenotyping

Another significant gap is the need for more research into the precise biological mechanisms by which ND-CNVs influence eating behaviours. While some initial associations between specific ND-CNVs and eating behaviours have been identified (72), the underlying pathways remain poorly understood. Research should focus on elucidating the molecular and cellular mechanisms involved, such as how ND-CNVs affect neural circuits regulating hunger and satiety, or how they influence metabolic pathways. Identification of variants allows for post-GWAS bioinformatic approaches and genetic-first studies of carriers, as well as rodent model studies. Deep phenotyping is crucial for understanding how eating behaviours and eating disorder sequelae co-develop prospectively across development. Initial rodent model work and zebrafish studies have investigated the impact of homologs of the 16p11.2 region on growth phenotypes (79, 80). To develop a comprehensive understanding of how ND-CNVs influence eating behaviour and eating disorder phenotypes, it will be essential to integrate genetic, neurobiological, and behavioural data to identify potential biomarkers for early detection and intervention. Network analysis and systems biology analytical approaches are needed to integrate data from multiple biological levels (81).

### 3.3 Diverse populations

The majority of research investigating the impact of ND-CNVs on eating disorder and eating behaviour traits have been conducted in populations of European ancestry, creating concerns that findings may not be applicable to a large fraction of the global population. Research involving cohorts from a range of populations is necessary to ensure that the associations identified are applicable across different ancestral and genetic backgrounds (6). There is clear evidence for common genetic risk factors that polygenic risk scores (PRS) developed from multi-ancestry genome-wide association studies improve predictive performance of PRS, and diversifying genomic studies is important step to achieving equitable PRS performance across ancestral populations (82). The importance of cohort ancestry when characterising the medical phenotypes of rare genetic variants is evident from recent work where ND-CNV prevalence and clinical phenotype differed between European-ancestry cohorts and a multi-ancestry cohort (BioMe) (83). However the authors of this study note that cohort differences at some CNV loci cannot be directly attributable to ancestry divergence, and may be partly attributable to systematic biases in CNV-calling algorithms (83), highlighting the need for analytical pipelines developed and trained on genetic data from a range of ancestries. The Psychiatric Genomics Consortium (PGC) is actively expanding its work across multiple ancestral populations (84) by leveraging diverse cohorts such as the new All of Us research program biobank (85) and collaborating with international psychiatric genetics initiatives such as the Ancestral Populations Network (86). The NeuroDev study is an example of research that is transforming insights into rare variants and neurodevelopmental conditions in an African context (87, 88).The NeuroDev study is conducting detailed phenotyping on cognition, behaviour, and medical traits in an expected cohort of 5,600 Africans (1,800 children with neurodevelopmental conditions, 1,800 child controls, and 1,900 parents) and is collecting blood samples for exome sequencing and biobanking, with preliminary with the first year of data analysed representing the first trio-based study of neurodevelopmental conditions in Kenya and South Africa (87). In terms of population cohorts, the Born in Bradford Age of Wonder study has introduced eating disorder measures following consultation with teenagers living in Bradford (89). Bradford has a multi-ethnic population with the census data from 2021 showing that 61% of the population identified as White British, 32% as Asian/Asian British, and 34% of Bradford residents live in areas that rank in the most deprived decile of local areas in England (90). The sociodemographic profile of the Born in Bradford study enables investigation of the genetics of eating disorders within groups previously underrepresented in research, and highlights the benefits of enriching existing cohorts across populations for eating disorder phenotype data.

### 3.4 Cross-disciplinary collaboration

Addressing the research gaps presented in this perspective will require collaboration between geneticists, neuroscientists, and clinicians, across research areas and clinical specialities. Studies of common genetic risk factors of AN implicate genes involved in the brain and also metabolic processes, highlighting the need for interdisciplinary work to translate these genetic findings into mechanistic and intervention research (55). Cross-disciplinary collaboration can foster innovative systems biology approaches and lead to more effective interventions. For example, initial brain imaging studies of 16p11.2 Deletion and Duplication have identified gene-dosage effects on white matter properties in cortico-subcortical regions implicated in reward processing (91). Further research is needed to understand how this relates to eating behaviour outcomes. Involving patients, families, and advocacy groups in research is crucial for ensuring that studies address the real-world needs and concerns of those affected. Community engagement can help researchers design studies that are relevant and meaningful to participants, improving recruitment and retention rates. Additionally, qualitative research can provide valuable insights into the lived experiences of individuals with rare variants. For example, a qualitative study into the experience of carers of children with 16p11.2 Deletion and Duplication variants issues surrounding metabolism and eating patterns represented one of the top themes of parental concern (92).

## 4 Future perspectives

### 4.1 Early identification and intervention

Advances in understanding the contribution of rare variants to eating disorders and eating behaviour has the potential to enable new research designs for examining early intervention for disordered eating. The identification of rare variants associated with eating disorders would enable genetic-first studies that prospectively examine the impact of genomic risk on early eating behaviour trajectories leading to disorder eating. Drawing parallels from the schizophrenia field, prospective developmental studies have been conducted across childhood and adolescence in individuals with 22q11.2 Deletion Syndrome (93–95), where 30-40% of individuals are at risk of developing psychosis in adulthood (96, 97). Longitudinal studies of individuals with 22q11.2 Deletion Syndrome have identified a range of developmental precursors for psychotic phenomena, including anxiety, Verbal Intelligence Quotient (VIQ) trajectory, ADHD symptoms, and executive functioning ability (94, 98–100). Work from the International Brain & Behaviour Consortium found that VIQ trajectories for those who later develop psychosis diverged from age 11 (93). This has led to a prospective neuroprotective clinical trial for psychosis in 22q11.2 Deletion Syndrome (101). Studies of children at high genomic risk would provide insights into early risk signs for eating disorders, allowing healthcare providers to intervene sooner and potentially mitigate the severity of eating disorders and their associated health consequences (102).

### 4.2 Tailored treatment approaches

Understanding the genomic underpinnings of eating disorders can enable genetic counselling approaches and the development of more effective, individualised treatment plans. For example, there are already clinics for ND-CNVs in Canada, such as the Developmental Assessment of Genetically Susceptible Youth (DAGSY) Clinic, a novel interdisciplinary ‘genetic-diagnosis-first’ clinic integrating psychiatric, psychological, and genetic expertise (103). There is also the All Wales Psychiatric Genomics Service, a collaborative effort between psychiatric and clinical genetics services and the first of its kind in the UK, whereby adults with complex psychiatric presentations can be referred for genetic testing and genomic counselling (104). Several healthcare systems have specialised clinics for specific CNVs, such as Prader-Willi Syndrome (105) and 22q11.2 Deletion Syndrome (106), that provide specialised care and have established syndrome registries for understanding natural history.

It is not known whether traditional treatments for eating disorders would be as effective for individuals with ND-CNVs and may require modification for various reasons, including developmental delay, sensory impairments, multimorbidity of physical health problems, and sensitivity to adverse effects. However, it is known that for other clinical features neurodevelopmental CNV carriers experience, treatment adaptations may be needed. For example, ND-CNV carriers who experience cognitive and social difficulties may find it challenging to access and benefit from therapies such as cognitive behavioural therapy. Adaptations to therapies, such as shorter sessions, frequent breaks, and repetition of content, should be considered (107). There is also evidence that ND-CNV carriers with autism benefit less from social skills training than autistic children but without a pathogenic CNV (108). By tailoring treatment approaches to the specific needs of children with ND-CNVs, healthcare providers can improve outcomes and enhance the overall effectiveness of interventions.

## 5 Conclusion

The exploration of rare ND-CNVs offers a promising avenue for understanding the genetic and biological underpinnings of eating disorders and related traits. While significant progress has been made in identifying the role of CNVs in psychiatric and neurodevelopmental conditions, their impact on eating behaviours remains underexplored. This perspective highlights the potential of ND-CNV research to provide transformative insights into the genetic architecture of eating disorders, as has been for other psychiatric and physical health conditions. The identification of ND-CNVs associated with eating disorders has the potential to facilitate early identification and intervention, leading to improved outcomes for affected individuals. However, current research is limited by lack of large-scale cohorts, phenotype bias, a lack of mechanistic insights, and insufficient diversity in study populations. Addressing these gaps requires cross-disciplinary collaboration and the integration of genetic, neurobiological, and behavioural data. By leveraging genetic-first studies and tailored treatment approaches, researchers and clinicians can enhance our understanding of the complex interactions between genetics, neurodevelopment, and eating behaviours. Ultimately, this knowledge will pave the way for more effective interventions and improved quality of life for individuals with ND-CNVs, contributing to personalised medicine approaches for eating disorders and the management of challenging eating behaviour.

## Data Availability

The original contributions presented in the study are included in the article/supplementary material. Further inquiries can be directed to the corresponding author.
